# Moving beyond response times with accessible measures of manual dynamics

**DOI:** 10.1038/s41598-022-20579-9

**Published:** 2022-11-09

**Authors:** Katie Ann Smith, Samara Morrison, Annette M. E. Henderson, Christopher D. Erb

**Affiliations:** School of Psychology, University of Auckland, 23 Symonds Street, Building 302, Auckland, 1010 New Zealand

**Keywords:** Psychology, Human behaviour

## Abstract

Button-press measures of response time (RT) and accuracy have long served a central role in psychological research. However, RT and accuracy provide limited insight into how cognitive processes unfold over time. To address this limitation, researchers have used hand-tracking techniques to investigate how cognitive processes unfold over the course of a response, are modulated by recent experience, and function across the lifespan. Despite the efficacy of these techniques for investigating a wide range of psychological phenomena, widespread adoption of hand-tracking techniques within the field is hindered by a range of factors, including equipment costs and the use of specialized software. Here, we demonstrate that the behavioral dynamics previously observed with specialized motion-tracking equipment in an Eriksen flanker task can be captured with an affordable, portable, and easy-to-assemble response box. Six-to-eight-year-olds and adults (*N* = 90) completed a computerized version of the flanker task by pressing and holding a central button until a stimulus array appeared. Participants then responded by releasing the central button and reaching to press one of two response buttons. This method allowed RT to be separated into *initiation time* (when the central button was released) and *movement time* (time elapsed between initiation and completion of the response). Consistent with previous research using motion-tracking techniques, initiation times and movement times revealed distinct patterns of effects across trials and between age groups, indicating that the method used in the current study presents a simple solution for researchers from across the psychological and brain sciences looking to move beyond RTs.

## Introduction

Button-press measures of response time (RT) and accuracy have served as cornerstones of quantitative research in psychology since the mid-twentieth century. However, such measures provide limited insight into how the processes underlying perception, cognition, and action unfold over time. Consequently, researchers have developed sophisticated computational^1–6^, neural^7–11^, and behavioral methods^12–17^ to shed light on how cognitive processes unfold over the course of a response (*within-trial dynamics*), are modulated by recent experience (*cross-trial dynamics*), and function across the life span (*developmental dynamics*).

**Figure 1:**
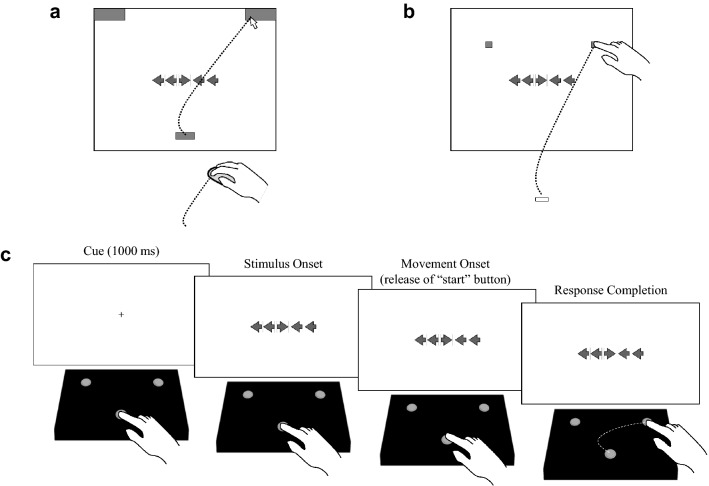
(**a**) Illustration of a mouse-tracking version of the flanker task. In the task, participants are instructed to respond according to a centrally presented target stimulus and to ignore surrounding distractor stimuli. Participants initiate each trial from a starting location at the bottom center of the display. Following stimulus presentation, participants navigate the mouse cursor to a response target located at the top of the screen. (**b**) Illustration of a reach-tracking version of the task. Participants initiate each trial by placing their finger on a designated starting marker place on the table in front of them. Following stimulus presentation, participants respond by reaching to touch a response target on the digital display. Hand movements can be recorded with optical tracking systems or electromagnetic tracking systems, both of which usually require the participant to wear a motion tracking sensor on their pointing finger. (**c**) Illustration of the button-release-and-press method used in the current study. Participants initiate each trial by holding down a “start” button at the bottom center of a response box. Following stimulus presentation, participants release the “start” button and respond by pressing one of two response buttons located toward the top of the response box. IT is measured as the time elapsed between stimulus presentation and the release of the “start” button, whereas MT is measured as the time elapsed between the release of the “start” button and the pressing of a response button. Portions of this figure were adapted from Erb^40^ with permission from the author.

The limitations of button-press measures of RT and accuracy are illustrated by recent research using *hand-tracking* techniques to investigate the dynamics of attention and control^18–22^. In contrast to standard button-press measures, techniques such as *mouse tracking* and *reach tracking* provide insight into the temporal and spatial dynamics of performance by recording the path that a participant’s hand travels to reach a response target (see Fig. 1a,b). For example, these techniques allow RT to be deconstructed into measures of *initiation time* (IT; the time elapsed between stimulus onset and movement initiation) and *movement time* (MT; the time elapsed between movement initiation and response completion).

Recent reach-tracking studies demonstrate that the patterns of effects observed in RTs in common congruency tasks such as the Eriksen flanker task^23^ reflect distinct patterns of effects in ITs and MTs^18,24,25^. In the flanker task, participants indicate which direction a centrally presented target arrow is pointing on each trial (see Fig. 1). On congruent trials, the target arrow is surrounded by distractor arrows that cue the same response (e.g., >>>>>). On incongruent trials, the distractor arrows cue a different response than the target arrow (e.g., <<><<). A *congruency effect* is standardly observed in the task, with higher error rates and slower response times on incongruent relative to congruent trials (I–C).

**Figure 2:**
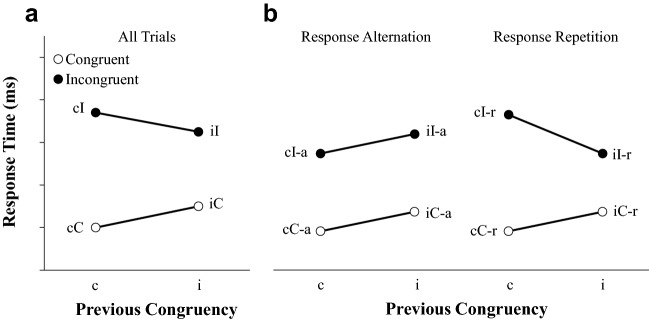
(**a**) Illustration of the congruency sequence effect (CSE) observed in response times (RTs) in a two-response version of the flanker task, with a smaller congruency effect on trials preceded by an incongruent trial (iI and iC trials) than on trials preceded by a congruent trial (cI and cC trials). (**b**) Illustration of the RT effects observed in the same task when trials featuring the opposite response of the preceding trial (response alternation trials) are separated from trials featuring the same response as the preceding trial (response repetition trials). Note that the CSE pattern is only observed in response repetition trials.

Additionally, a *congruency sequence effect* (CSE) (or *Gratton effect*)^26^ is frequently observed in the flanker task, with a larger congruency effect observed in trials preceded by a congruent trial (cI–cC, where the lowercase letter denotes the congruency of the previous trial) relative to trials preceded by an incongruent trial (iI–iC) (see Fig. 2a). Multiple studies have found that the CSE observed in two-response versions of the flanker task is driven by *response repetition trials* in which the response of the previous trial is required on the current trial (see Fig. 2b)^24,27,28^. In contrast, RTs on *response alternation trials* have been found to feature main effects of the current trial’s congruency (slower on incongruent trials than congruent trials) and the previous trial’s congruency (slower on trials preceded by an incongruent trial relative to trials preceded by a congruent trial).

**Figure 3:**
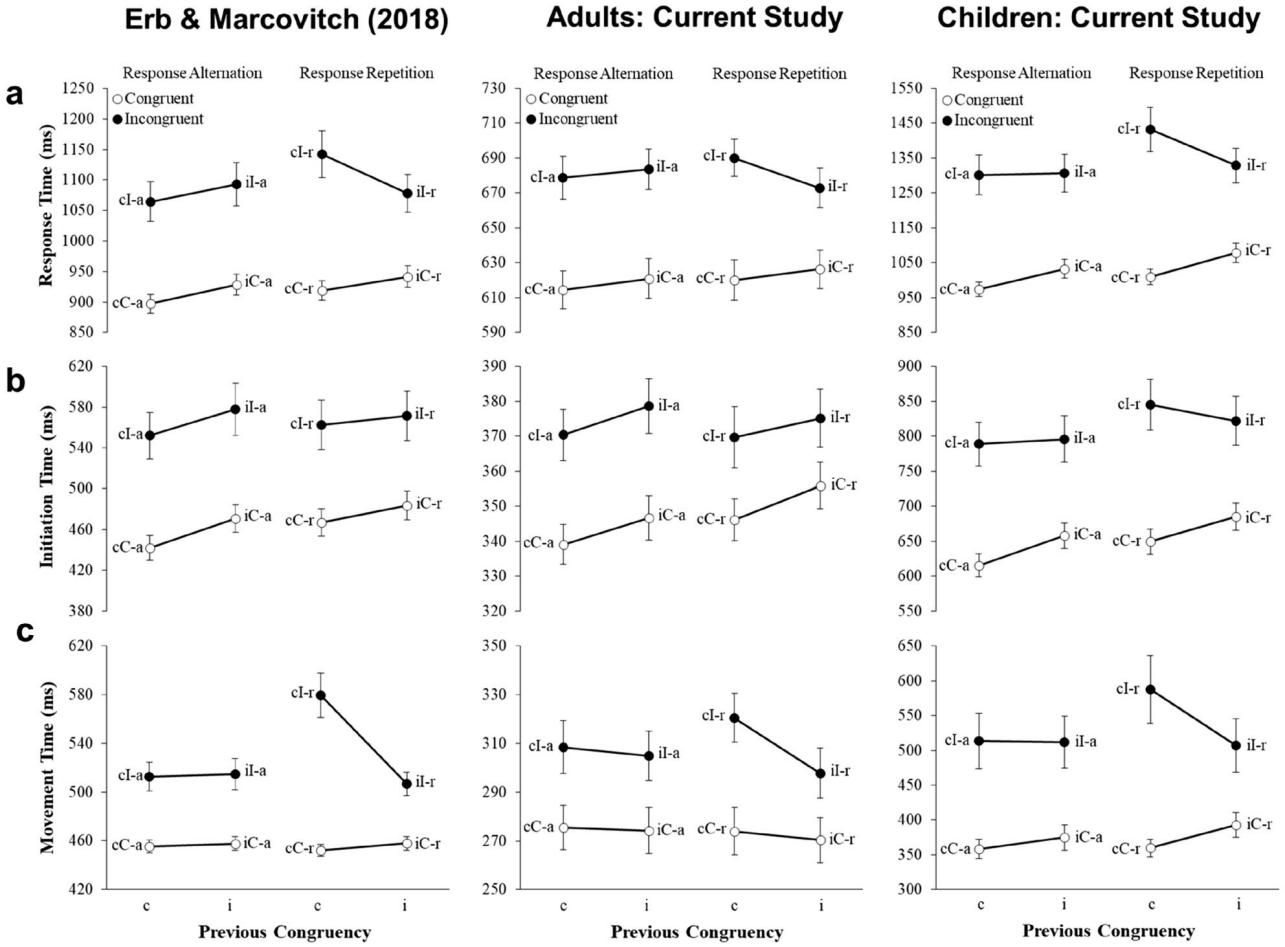
(**a**) Response time (RT), (**b**) initiation time (IT), and (**c**) movement time (MT) data from a two-response version of the flanker task with 135 participants (45 6- to 8-year-olds, 45 10- to 12-year-olds, and 45 adults) by Erb and Marcovitch^24^ collected using an electromagnetic reach-tracking system (left panel) and data obtained in the current study using a button-release-and-press method from adult participants (middle panel) and 6- to 8-year-old participants (right panel). Error bars denote standard errors. Data obtained by Erb and Marcovitch^24^ presented with permission from the authors.

Reach tracking investigations of the flanker task demonstrate that the CSE observed in RTs in two-response versions of the task (see the left panel of Fig. 3a) reflects the combination of two distinct patterns of effects: one observed in ITs and another observed in MTs^18,24^. Specifically, ITs reveal a main effect of current and previous congruency in response alternation and response repetition trials (see the left panel of Fig. 3b), whereas MTs reveal a main effect of current congruency in response alternation trials and a CSE in response repetition trials (see left panel of Fig. 3c). Erb et al.^19^ suggested that the patterns of effects observed in ITs and MTs reflect two dissociable processes underlying cognitive control: a *threshold adjustment process* involving the global inhibition of motor output in response to signals of conflict^29–34^, and a *controlled selection process* that regulates top-down attentional resources to bias competing response activations towards the correct response, respectively^35,36^. Thus, in addition to demonstrating that RT patterns commonly obscure underlying effects of interest^15^, collecting separate measures of IT and MT presents new opportunities to clarify how the processes that underlie decision behavior unfold over time in different tasks, age groups, and individuals^24,25,37–41^.

Although measuring the temporal and spatial characteristics of hand movements (i.e., *manual dynamics*) has proven instrumental to studying topics across perceptual, cognitive, social, and developmental psychology^17–22,42–57^, hand-tracking techniques remain comparatively rare in psychological research relative to standard button-press measures. This reliance on button-press measures can be attributed to a range of factors, including the cost and lack of portability associated with optical or electromagnetic motion-tracking systems, the use of specialized software, and ongoing discussions concerning the analysis and interpretation of hand-tracking data^58–61^.

In contrast to optical or electromagnetic motion-tracking systems, mouse tracking presents an easy to use, affordable, and portable solution for researchers who want to measure manual dynamics^13,14,53,56,59^. However, the extent to which mouse tracking can be used to capture the patterns of effects observed in ITs and MTs in reach-tracking studies remains unclear. This lack of clarity is partly because most mouse-tracking studies have used a dynamic starting procedure in which participants initiate their movement before the operative stimulus is presented, thereby preventing measurement of IT^13,62^. As such, ITs in tasks featuring dynamic starting procedures are commonly used to define exclusion criteria in mouse tracking paradigms, but are not commonly analyzed.

A recent mouse-tracking study by Ye and Damian^63^ used a static starting procedure in which participants initiated their movements after the operative stimulus was presented in three different congruency tasks, including the flanker task. Consistent with the reach-tracking studies reviewed above, the researchers observed a main effect of previous congruency, with slower initiation times on trials preceded by an incongruent as opposed to a congruent trial. However, no effect of current congruency was observed in initiation times, suggesting that mouse tracking may be less effective at capturing the different patterns of effects observed in ITs and MTs in reach-tracking tasks. Additionally, it is unclear how effective mouse-tracking tasks are for gathering data from children, given that children can vary greatly with regard to their skills with a computer mouse^64^. This consideration is especially relevant given that mouse-tracking studies require participants to perform visuomotor transformations to link their hand movements to the movements of a mouse cursor^21^.

In light of (a) the substantial barriers to adopting optical or electromagnetic motion-tracking techniques and (b) the potential limitations of mouse tracking outlined above, the current study investigates the extent to which the within-trial, cross-trial, and developmental dynamics observed with specialized hand-tracking systems^24^ can be captured using a button-release-and-press method in which participants respond by releasing a “start” button and then pressing one of two response buttons (see Fig. 1c)^65–69^. Demonstrating that this method can be used to capture the behavioral dynamics observed with more specialized systems would provide a simple, accessible solution for researchers across the behavioral and brain sciences looking to move beyond RTs.

## Method

### Preregistration and data availability

This study was preregistered through the Open Science Framework website on September 23rd, 2019 using the AsPredicted.org template (https://osf.io/bqsf3). We present our preregistration along with accompanying comments in Sect. 1 of the Supplementary Materials. The data and analysis files for the current study are available at https://osf.io/zpyvf/?view_only=761bd50ec9714aaba6b6fcc2101651ff. As detailed in the preregistration, the current study also collected eye-tracking data. For the purposes of this report, we focus on the hand movement data and report the full set of preregistered analyses (including analyses related to eye movements) in Sect. 2 of the Supplementary Materials.

### Participants

The final sample consisted of 15 6-year-olds (*M* = 77.1 months, *SD* = 3.0, 8 female), 15 7-year-olds (*M* = 88.5 months, *SD* = 2.7, 8 female), 15 8-year-olds (*M* = 101.0 months, *SD* = 3.5, 5 female) and 45 adults (*M* = 20.7 years, *SD* = 2.0, 27 female). To be eligible for the study, participants had to be right-handed, have normal or corrected-to-normal vision, understand English, be able to perform normal reaching movements, and have no diagnosis of a social or cognitive impairment. Adults provided informed consent prior to testing. Child participants provided their assent, and informed consent was obtained from a legal guardian prior to testing. One additional adult was tested but excluded from the final dataset due to a software error. Three additional children were tested but excluded from the final dataset due to failure to complete the task. Adults received course credit or $15 NZD for participating. Child participants were compensated with a small prize worth approximately $5 (e.g., a plush toy). The families of participating children were also entered into a drawing for the chance to win a $25 supermarket voucher. Testing was conducted at the University of Auckland City campus and the study was approved by the University of Auckland Human Participants Ethics Committee (UAHPEC). Testing was performed in accordance with APA Ethical Principles.

### Apparatus

The task was displayed using a 53 cm × 29.6 cm digital monitor. Participants were seated at a small table facing the monitor. Participants made their responses using three buttons (3.0 cm in diameter) located toward the top left, top right, and bottom center of a 36.0 cm × 23.0 cm response box positioned centrally on the table in front of them (see Fig. 1c). Section 3 of the Supplementary Materials provides details regarding the construction of the response box, including instructions for researchers interested in constructing a custom response box. The task was designed and run using E-Prime^®^ 3.0.

### Procedure

Participants completed a computerized two-response version of the Eriksen flanker task featuring arrow stimuli (see Fig. 1). Participants initiated each trial by pressing and holding a central “start” button. After 1 s, the stimulus array appeared. Participants then made their response by releasing the “start” button and reaching to press either the right or left response button. Participants in all age groups were first given a verbal explanation of the task instructions. Child participants were shown a printed picture of the four possible stimulus arrays and were asked to demonstrate (using the response box) which response was correct in each case. The experimenter demonstrated the procedure for adult participants in the first two trials of the practice block. Participants then completed a block of 16 practice trials. During the practice trials, the experimenter (seated next to the participant) provided guidance and corrective instructions to participants in all age groups (e.g., “remember to look at the middle arrow” or “keep holding the start button down until the arrows appear”). A high tone (600 Hz for 200 ms) followed correct responses and a low tone (300 Hz for 200 ms) followed incorrect responses. There was no time limit for responses in the practice trials.

Adults completed the experimental blocks with a black curtain drawn between them and the experimenter. This curtain was left open for child participants after pilot testing revealed the need for continued monitoring and scaffolding from the experimenter (e.g., “remember to only press the buttons with your right hand”). Participants were presented with four blocks of 48 trials, for a total of 192 experimental trials. Twenty-one adult participants and 13 child participants were presented with 41 trials in the third block due to a coding error. Each block consisted of an equal number of congruent and incongruent trials, and an equal number of trials wherein the target arrow cued the left and right responses. Trial order was randomized for each block of trials. If the participant provided an incorrect response or took longer than 4 s to respond, the trial ended, and a low tone sounded. Correct responses in the experimental trials were not followed by any feedback. Participants were instructed to respond as quickly and accurately as possible.

At the end of each experimental block, the word “Rest” appeared in the centre of the screen. For adult participants, the experimenter verbally confirmed with the participant that they were ready to start the next block after approximately 5 s. Child participants were given the opportunity to take a break after each experimental block. The experimenter initiated the next block after the child confirmed that they were ready to continue. Trials containing an invalid response (e.g., the participant pressed the “start” button instead of the left or right button after stimulus presentation) and trials in which no response was provided within the 4 s limit, were excluded from analysis. Error rate, RT, IT and MT measures for each trial were obtained directly from E-prime^®^ outputs. IT was defined as the time elapsed between the appearance of the stimulus array and release of the “start” button. MT was defined as the time elapsed between release of the “start” button and response completion. RT was computed for each trial by summing IT and MT.

## Results

The first trials of each block was excluded from all analyses. Error rates were at floor for adult participants (*M* = 0.47%, *SD* = 0.01). Child participants made significantly more errors than adults [*M* = 4.96%, *SD* = 0.07, *F*(1, 88) = 18.51, *p* < .001]. Error rates for child participants also revealed a significant congruency effect *F*(1, 44) = 11.31, *p* = .002, with more errors in incongruent trials (*M* = 7.81%, *SD* = 0.13) relative to congruent trials (*M* = 2.18%, *SD* = 0.03). Trials featuring an error, and trials preceded by an error were excluded from the subsequent analyses. Log transformations were applied to RT, IT, and MT to minimize the effect of processing speed differences between children and adults. These measures were then analyzed using mixed analyses of variance (ANOVAs), featuring age group (children, adults) as a between-subjects factor, and previous congruency (c, i), current congruency (C, I), and response type (repetition, alternation) as within-subjects factors. Bonferroni corrections were applied to all post-hoc analyses to correct for multiple comparisons. Results of ANOVAs performed on log transformed and raw data are available in Sects. 4 and 5 of the Supplementary Materials, respectively. Effect sizes in the figures and text below is presented in milliseconds (without the log transformation) to aid interpretability.

In keeping with previous findings, RTs revealed a significant CSE [58 ms; *F*(1,88) = 32.08, *p* < .001, η_*p*_^2^ = 0.27], with a larger congruency effect on trials preceded by a congruent trial (220 ms) than trials preceded by an incongruent trial (162 ms). The CSE was specific to response repetition trials [98 ms; *F*(1,88) = 38.02, *p* < .001, η_*p*_^2^ = 0.30], with response alternation trials revealing main effects of current congruency [186 ms; *F*(1,88) = 133.34, *p* < .001, η_*p*_^2^ = 0.60] and previous congruency [17 ms; *F*(1,88) = 12.12, *p* < .001, η_*p*_^2^ = 0.12] but no interaction between the two, consistent with previous research (see Fig. 3a)^24,27,28^.

The overall pattern observed in RTs reflected the sum of two distinct trial sequence effects in ITs and MTs. Specifically, ITs revealed main effects of current congruency [94 ms; *F*(1,88) = 161.74, *p* < .001, η_*p*_^2^ = 0.65] and previous congruency [10 ms; *F*(1,88) = 23.14, *p* < .001, η_*p*_^2^ = 0.21; see Fig. 3b]. This pattern of effects has been previously proposed to reflect a threshold adjustment process in which signals of conflict lead to higher response thresholds and, consequently, longer initiation times on incongruent trials and trials following an incongruent trial^24^. MTs revealed a significant interaction among current congruency, previous congruency, and response repetition type [*F*(1,88) = 18.59, *p* < .001, η_*p*_^2^ = 0.17]. Follow-up tests revealed a main effect of current congruency on response alternation trials [91 ms; *F*(1,88) = 70.92, *p* < .001, η_*p*_^2^ = 0.44]. In contrast, response repetition trials revealed a significant interaction between current and previous congruency [*F*(1,88) = 45.88, *p* < .001, η_*p*_^2^ = 0.34]. Follow-up tests revealed slower MTs on cI-r trials (454 ms) than iI-r trials (402 ms), with a significantly smaller difference between cC-r (317 ms) and iC-r (332 ms) trials (see Fig. 3c; *t*(89) = 6.77, p < .001). Erb and Marcovitch^24^ also observed this pattern of effects in MTs and attributed the difference between cI-r and iI-r trials to feature-integration effects occurring when the stimulus and response features of the previous trial partially overlap (cI-r trials) or fully overlap (iI-r trials) with those of the current trial^28,70,71^.

Several developmental differences of note were also observed. The congruency effect observed in RTs decreased significantly with age [children: 323 ms; adults: 61 ms; *F*(1,88) = 46.02, *p* < .001, η_*p*_^2^ = 0.34; see Fig. 3a], with a significant age-related decrease in both ITs (children: 162 ms; adults: 27 ms; see Fig. 3b) and MTs (children: 161 ms; adults: 35 ms; see Fig. 3c). The size of the CSE also significantly decreased with age in both RTs [children: 104 ms; adults: 12 ms; *F*(1,88) = 12.72, *p* = .001, η_*p*_^2^ = 0.13] and MTs [children: 61 ms; adults: 11 ms; *F*(1,88) = 7.36, *p* = .008, η_*p*_^2^ = 0.08]. Children’s performance in the current study diverged from the findings of Erb and Marcovitch^24^ in two ways. Firstly, children’s ITs in the current study revealed a CSE [*F*(1,44) = 10.14, *p* = .003, η_*p*_^2^ = 0.19], and did not reveal an effect of previous congruency on incongruent trials, indicating that patterns in ITs and MTs are less distinct in children than adults. Secondly, we did not observe the four-way interaction in MTs reported by Erb and Marcovitch^24^ in which age-related decreases in the CSE were specific to response repetition trials.

## Discussion

The results of the current study demonstrate that a simple button-release-and-press method can be used to capture distinct patterns of effects in ITs and MTs that are otherwise obscured in RTs. Specifically, RTs revealed main effects of current and previous congruency in response alternation trials and a CSE in response repetition trials, consistent with previous reach-tracking and button-press research^24,27,28^. The pattern observed in RTs was demonstrated to reflect distinct patterns of effects in ITs and MTs, consistent with previous work indicating that these measures can be used to capture the functioning of the threshold adjustment process and controlled selection process, respectively^18,24^. Thus, our findings indicate that a simple, affordable, and portable response box can be used to capture distinct patterns of effects in RTs, ITs, and MTs in a manner comparable to specialized optical or electromagnetic tracking systems.

In contrast to the reach tracking results from Erb and Marcovitch^24^, the button-release-and-press method used in the current study proved to be less effective for capturing distinct effects in ITs and MTs in children. We suspect that this difference emerged because children in the current study often looked down to the response box before initiating their response, possibly delaying their movement and resulting in a blending of the typical IT and MT patterns. This possibility is supported by the observation that children spent a smaller proportion of time during experimental trials with their eyes on-screen (*M* = 0.86, *SD* = 0.25) than adults (*M* = 0.99, *SD* = 0.05). To address this, future studies may incorporate additional practice using the button box for child participants prior to experimental trials. Alternatively, to reduce the possibility of split attention and to make the procedure more similar to past hand-tracking research with children, future studies could have children respond by reaching to images on a touchscreen display instead of separate buttons.

Although the patterns of effects observed in adults in the current study correspond closely to the patterns observed in previous research using electromagnetic motion-tracking systems, the strongest demonstration of the button-release-and-press method would come from a study in which the same group of participants complete the same tasks with both methods. As noted in the “Introduction”, it is not clear at present whether mouse tracking can be used to deconstruct RTs in the same manner as the button-release-and-press method tested in the current study^63^. Consequently, future research should directly compare mouse tracking, 3-dimensional reach tracking, and the button-release-and-press method in tasks targeting attention and control.

A recent study by Moher and Song^72^ compared 3-dimensional reach tracking, mouse tracking, and stylus tracking in a task in which a target stimulus would occasionally change locations after the participated initiated a movement. The researchers found that the three methods generated results that were largely comparable. However, hand movements measured with the 3-dimensional reach-tracking system revealed (a) faster initiation latencies than movements measured with a stylus and (b) more curved movement trajectories than movements measured with a stylus or computer mouse. These observations led Moher and Song to conclude that, “3D reach trackers may be ideal for observing fast, subtle changes in internal decision-making processes compared to other devices” (p. 2558). Although the button-release-and-press method used in the current study does not capture the spatial dynamics of responding, the results of Moher and Song suggest that 3-dimensional hand movements like those required in the current study may be particularly useful for capturing initiation time effects.

Our findings present important implications for research across the behavioral and brain sciences that collects button-press measures of RT. For example, many of the standardized cognitive assessments used to measure developmental and individual differences^73^ could incorporate a button-release-and-press method to collect measures of IT and MT. This approach also presents an alternative solution to the challenges of interpreting developmental and individual differences in performance when speed-accuracy trade-off effects may be present^74,75^ given that the method allows participants to detect and override incorrect responses, resulting in very low error rates.

This approach can also be easily paired with electroencephalography (EEG) to shed new light on the link between the brain and behavior. Electromagnetic position and orientation recording systems such as those used by Erb and Marcovitch^24^ are sensitive to ferromagnetic materials and may therefore be difficult to pair with EEG. Although researchers have incorporated more continuous measures of behavior into EEG studies using electromyography (EMG)^8,76^ mouse tracking^77^, and dynamometers^78,79^, the neural correlates of the threshold adjustment and controlled selection processes captured with reach tracking are currently unclear. The button-release-and-press method used in the current study is compatible with any equipment that can be used with a standard keyboard, including EEG. Consequently, our research group is currently using the approach to target neural activity preceding movement initiation in EEG investigations of the flanker^23^ and Simon tasks^80^.

## Conclusion

RTs have long served as a foundational behavioral measure in the psychological and brain sciences. Although the current study focused on the dynamics of cognitive control, the ability to deconstruct RTs into ITs and MTs could provide important insights into the cognitive processes underlying a wide range of phenomena, including perception, memory, language, numerical cognition, social reasoning, and decision making. Additionally, the release-and-press method used in the current study can be paired with other techniques such as eye tracking and EEG to shed new light on how manual, oculomotor, and neural dynamics are linked^81,82^. Although hand-tracking techniques have demonstrated great potential for studying the mind in action^17–22,42–57^, these techniques have yet to be widely adopted within psychological research. The results of the current study demonstrate that a simple, portable, and low-cost button-release-and-press method can provide a robust, accessible solution for researchers looking to move beyond response times.

## Supplementary Information

Supplementary Information.
