# Supplementary material for: Moving beyond response times with accessible measures of manual dynamics
Source: Sci Rep. 2022 Nov 9;12:19065. doi: 10.1038/s41598-022-20579-9 (PMC9646795; doi:10.1038/s41598-022-20579-9)
Supplement: Supplementary file 1 — Supplementary Information. [file 41598_2022_20579_MOESM1_ESM.pdf]

Supplementary Materials

Section 1: Preregistration an accompanying comments

This project was preregistered on the Open Science Framework website on September 23rd, 2019 using the AsPredicted.org template. In the following, we present our preregistration with accompanying comments noting any deviations from the preregistration. Our responses to the questions are presented in italics and our comments are presented in bold text. The preregistration is available at https://osf.io/bqsf3.

Description

Recent research using motion-tracking equipment indicates that the spatial and temporal dynamics of hand movements (i.e., manual dynamics) can be used to investigate how processes underlying cognitive control unfold in congruency tasks like the Eriksen flanker task (Eriksen & Eriksen, 1974). For instance, Erb and Marcovitch (2018) found that movement initiation times (the time elapsed from stimulus presentation to movement initiation) revealed a different pattern of trial sequence effects than movement times (the time elapsed from movement initiation to response completion) and reach curvatures (a measure of the extent to which a movement trajectory deviated from a direct path to the selected response target). Further, the researchers observed different developmental trajectories in these measures, indicating that key processes underlying cognitive control develop at different rates. The current study will address three main questions. Q1. Can the manual dynamics observed with motion-tracking equipment be captured using more portable and cost-effective equipment? The electromagnetic position and orientation recording system used by Erb and colleagues (Erb & Marcovitch, 2018; Erb, Moher, Sobel, & Song, 2016) is not portable and is relatively expensive. The equipment is also sensitive to ferromagnetic metals, making certain types of data collection difficult (e.g., gathering eye-tracking and hand-tracking data simultaneously). Thus, a primary aim of this project is to identify whether a button-release-and-press method can be used to

capture the manual dynamics observed with the electromagnetic position and orientation recording system. To that end, we developed a response box featuring a "home" button at the bottom centre of the box and two "response" buttons located toward the top corners of the box. To begin each trial, participants will press the "home" button. After the imperative stimulus array is presented, participants will release the "home" button and press one of the two "response" buttons. This will enable us to measure movement initiation time (the time elapsed between stimulus presentation and when the "home" button is released), movement time (the time elapsed between when the "home" button is released and when a "response" button is pressed), and response time (the time elapsed between stimulus presentation and when a "response" button is pressed). We will also use a video camera mounted over the response box to record hand movements. Our goal is to use a software package (e.g., Kinovia or OpenPose) to extract movement trajectories for each trial and then use these trajectories to compute curvature values in the same manner as Erb and colleagues (Erb & Marcovitch, 2018; Erb et al., 2016). To the extent that the manual dynamics observed with this button-release-and-press technique match those observed with less portable and less cost-effective motion-tracking techniques, the button-release-and-press approach might serve as an effective technique for researchers looking to incorporate measures of manual dynamics into their research. For example, the technique could be especially helpful for researchers conducting research outside of laboratory settings (e.g., in museums or schools). The suitability of this method as a replacement for less portable and less affordable motion-tracking techniques also depends on the population being evaluated. Given that the hand-tracking system used by Erb and colleagues appears to work well with children, we were particularly interested in evaluating whether the button-release-and-press technique would work well with children. Q2. How are oculomotor and manual dynamics related in performance of the flanker task? One of the benefits of using the button-release-and-press approach is that the technique can be paired

with eye-tracking equipment readily. To the extent that participants' reach movements do not interfere with a screen-mounted eye-tracking system's ability to record eye movements, we should be able to simultaneously measure oculomotor and manual dynamics. This could be particularly helpful for investigating differences in how individuals perform on cognitive tasks. For example, measuring oculomotor dynamics will enable us to evaluate whether individuals are preparing for an upcoming stimulus by fixating on a central cue, as well as the extent to which their gaze is captured by distractor stimuli. Investigating the extent to which oculomotor dynamics show similar patterns to manual dynamics will provide valuable insights regarding similarities and/or differences in the mechanisms and processes underlying cognitive functioning. Q3. To what extent can the developmental differences captured with motion-tracking equipment be captured with a button-release-and-press method? And, to what extent do these individual differences reflect different oculomotor dynamics? Previous research by Erb and Marcovitch (2018) revealed age-related gains in flanker task performance between middle childhood and adulthood in initiation times, movement times, response times, curvatures, and error rates. This study will evaluate the extent to which these developmental differences are observed with a button-release-and-press method. The study will also explore the extent to which the age-related gains in flanker performance might reflect a transition occurring during middle childhood from a more reactive style of control to a more proactive style of control (Chevalier, 2015, Child Development Perspectives). For example, in the flanker task, children who adopt a more reactive style of control may not adequately prepare for the upcoming trial by maintaining a constrained focus on the cue that appears before the stimulus array or the target arrow that appears in the stimulus array surrounded by distractor arrows. One way to assess this possibility is by measuring looking behaviour and assessing the extent to which looking behaviour in the flanker task differs across development. Similarly, at an

individual differences level, one could also evaluate whether individuals who do maintain a constrained focus on the cue and target outperform those who do not.

Hypotheses

Hypotheses regarding Q1: If the button-release-and-press technique is capable of capturing the same manual dynamics observed in reaching behaviour, then performance on the flanker task should replicate the patterns of trial sequence effects observed in initiation time, movement time, response time, and curvature by Erb and Marcovitch (2018).

Hypotheses regarding Q2 and Q3: If the button-release-and-press technique is capable of capturing the same developmental dynamics observed in reaching behaviour, then performance on the flanker task should reveal similar age-related differences to those observed between children and adults by Erb and Marcovitch (2018). If the age-related gains in flanker performance observed across childhood and between childhood and adulthood reflect a transition from a more reactive mode of control to a more proactive form of control, we should observe age-related differences in eye fixations during the presentation of the cue and during the presentation of the target and distractors. Specifically, children should fixate on the cue and target less than adults and should fixate on task irrelevant features (e.g., distractors or other parts of the screen) more.

Design Plan

Study type

Experiment - A researcher randomly assigns treatments to study subjects, this includes field or lab experiments. This is also known as an intervention experiment and includes randomized controlled trials.

Blinding

No blinding is involved in this study.

Is there any additional blinding in this study?

No response

Study design

The study will be a mixed design. The within-subject factors will be the congruency of the current trial (2; congruent vs. incongruent), the congruency of the previous trial (2; congruent vs. incongruent), and the response-repetition type of the current trial (2; Does the response generated on the current trial repeat or alternate relative to the previous trial?). The between-subjects factor will be age group (2; children vs. adults).

Randomization

Participants will not be assigned randomly to specific conditions. The presentation of trials will be randomized for each participant, meaning that one of four stimulus arrays (congruent with left response, congruent with right response, incongruent with left response, incongruent with right response) will be randomly presented on each trial, with each block consisting of an equal number of each stimulus array.

Sampling Plan

Existing Data

We are currently in the process of collecting data for the study but we have not yet processed or analysed any of the collected data. At of the time of this pre-registration, we have collected 11 child participants and 8 adult participants.

Data collection procedures

Population: *We are recruiting child participants (6 to 8 years of age) through a database of families who have expressed interest in having their children participate in research studies. The database is maintained by the Early Learning Lab at the University of Auckland. Participating families have their parking reimbursed and children receive a small gift for*

participating (valued around $5 NZD). Families will also be put into a drawing to win a $25 NZD gift certificate, with approximately 1 out of every 8 families receiving a gift certificate. We are recruiting adult participants (16 to 25 years of age) through a research participation pool maintained by the School of Psychology at the University of Auckland and through flyers posted around campus. Adult participants either receive course credit or $15 NZD for participating. To be eligible to participate, individuals must (a) be right-handed (or predominantly use their right-hand for reaching tasks), (b) have normal or corrected-to-normal vision, (c) understand English, and (d) have not been diagnosed with a social or cognitive impairment.

Task: *Participants will perform a version of the Eriksen flanker task based off of the task used by Erb and Marcovitch (2018, Cognition). Participants will first undergo a calibration phase with the eye-tracking system. Next, an experimenter will describe the rules of the flanker task and introduce participants to the button-release-and-press method. Participants will complete a set of practice trials. Following the practice trials, participants will be presented with 4 blocks of 48 trials. These blocks will consist of 24 congruent trials and 24 incongruent trials. Half of the trials will require a left response, whereas the other half will require a right response. The presentation of trials will be randomised in each block. During performance of the task, hand movements will be video recorded from a camera mounted over the response box. After the flanker task is complete, child participants will perform a standardised version of the Heads, Toes, Knees, and Shoulders (HTKS) task (Ponitz, McClelland, Jewkes, Connor, Farris, & Morrison, 2008).*

Sample size

Our target sample size is 90, with 45 child participants and 45 adult participants.

Sample size rationale

Our sample size for each age group was selected to match that of Erb and Marcovitch (2018, Cognition).

Stopping rule

We will stop data collection once we have collected usable data from 45 child participants and 45 adult participants (see Data Exclusion and Missing Data sections for details regarding what data will count as usable). If, during the course of processing and analysing the data, we discover an issue with the task, program, or equipment that substantially impairs our ability to address the three research questions of interest, we will stop data collection.

Variables

Manipulated variables

We will have three within-subjects factors: (1) the congruency of the current trial (C vs. I), (2) the congruency of the previous trial (c vs. i), and (3) the response repetition type of the current trial (response repetition vs. response alternation). We will have one between-subjects factor: (1) age group (children vs. adults).

Measured variables

Manual Measures: (1) Response time (time elapsed from stimulus presentation to response completion) (2) Initiation time (time elapsed from stimulus presentation to the release of the "home" button) (3) Movement time (time elapsed from the release of the "home" button until response completion) (4) Curvature (a ratio score reflecting the degree to which a movement trajectory deviated from a direct path to the selected target). Note: we will be attempting to use software packages to extract the spatial characteristics of hand movements from video files. It is presently unclear how effective this approach to computing reach curvatures will be. It is also unclear how much time will be needed to extract the relevant parameters. It is therefore possible that this measure will not be computed and analysed.

Note: Our attempts to extract the spatial characteristics of hand movements from the video files was unsuccessful, due to difficulties with automatising the process of identifying the location of the hand in each frame.

Oculomotor measures: (1) Cue fixation proportion score: The number of samples with fixations on the cue divided by the total number of samples gathered during the cue presentation period. In other words, for each trial, we will be identifying how many of the collected fixation measurements were on the cue. If fixation data were unavailable for samples (e.g., due to dropped samples or the participant looking off screen), these samples will not contribute to the proportion score. (2) Target fixation proportion score: The number of samples with fixations on the target divided by the total number of samples gathered during the presentation of the target and distractors. In other words, for each trial, we will be identifying how many of the collected fixation measurements were on the target during the presentation of the target and distractor items. If fixation data were unavailable for samples (e.g., due to dropped samples or the participant looking off screen), these samples will not contribute to the proportion score. (3) Distractor fixation proportion score: The number of samples with fixations on any of the distractors divided by the total number of samples gathered during the presentation of the target and distractors. In other words, for each trial, we will be identifying how many of the collected fixation measurements were on one of the distractors during the presentation of the target and distractor items. If fixation data were unavailable for samples (e.g., due to dropped samples or the participant looking off screen), these samples will not contribute to the proportion score.

Indices

We will be computing mean performance for each trial type and for each participant in the same manner as Erb and Marcovitch (2018, Cognition).

Analysis Plan

Statistical models

With regard to our measures of hand movements and eye fixations, our analytic strategy will mirror that of Erb and Marcovitch (2018, Cognition). We will use ANOVAs to evaluate the effects of Age Group, Current Congruency, Previous Congruency, and Response Repetition Type on the measures outlined above. We will follow-up on interaction effects in the same manner as Erb and Marcovitch (2018, Cognition) (note: the primary difference will be that Erb and Marcovitch collected data from three age groups, whereas the current study will collect data from two age groups). In addition to these analyses, we will evaluate the correlation between our oculomotor measures and age in months for our child participants to evaluate whether eye movements show evidence of a shift from a more reactive mode of control to a more proactive mode of control. We will also evaluate the correlation between our oculomotor measures and the size of the congruency effect observed in response times for our child participants. Finally, we will evaluate the correlation between the size of the congruency effect and performance on the HTKS task for child participants in order to identify the extent to which the measures might be tapping similar inhibitory control abilities.

Transformations

Following Erb and Marcovitch (2018, Cognition), we will log-transform the initiation time, movement time, response time, and reach curvature data. We will also run our analyses with the non-transformed data and make the results of those analyses available either in the main text or supplementary materials of any resulting publication. We will not log-transform the eye movement data.

Inference criteria

Following Erb and Marcovitch (2018, Cognition), we will set our alpha value to .05 for all analyses. All analyses will be two-tailed. Bonferroni corrections will be applied to adjust for multiple comparisons in both sets of correlational analyses.

Data exclusion

To be included in the final sample, participants will need to have completed (and have usable data from) at least 3 out of the 4 blocks of experimental trials. For all analyses, the first trial of each block will be excluded. For all analyses except for our analysis of error rates, only trials featuring an accurate response and following an accurate response will be included.

Missing data

We anticipate that eye movement data will not be available for each trial, as participants (particularly child participants) often move their head out of the region monitored by the eye tracker or obscure their eyes (e.g., by resting their head in their hand). If a participant's eye movement data are not available for at least 50% of the completed experimental trials, the eye tracking data for that participant will not be included in our analyses. Note that the participant's behavioural data will still be analysed, even if their eye movement data are not. In other words, the participant will still count towards the target sample size of 45 participants in each age group. In the event that the video of a participant's reaching behaviour is unavailable (e.g., due to technical difficulties), the participant's behavioural data (not including curvature) will still be analysed as well.

Exploratory analysis

We may perform exploratory analyses regarding pupillometry measures. As part of this project, we are also testing a smaller sample of 5-year-olds. In pilot testing, we found that 5-year-olds had a difficult time completing three or more blocks of the experimental trials. However, we are interested in evaluating 5-year-olds' performance on the task, particularly

with regard to their looking behaviour. We will therefore analyse 5-year-olds' data (assuming that at least two blocks of experimental trials have been completed) in the same manner as older children's and adults'. Finally, we will explore performance on trials featuring an error to identify, for example, whether fixation patterns differed on error trials relative to non-error trials. For instance, were fixations on the cue lower on error trials relative to non-error trials? Were fixations on the distractors higher?

Note: In the subsequent supplementary materials, we will not include data collected from the 5-year-old sample, in-keeping with the results reported in the current manuscript.

Section 2: Pre-registered analyses

In-keeping with our pre-registration, the following analyses pertaining to eye movement include data from participants with available eye movement data for over 50% of the experimental trials. Five children (two 6-year-olds, one 7-year-old, and two 8-year-olds) did not meet this criterion and were excluded accordingly.

2.1 Fixation proportion scores

Analysis of cue-, target-, and distractor-fixation proportion scores revealed increased fixations to task-relevant information. Adults spent a greater proportion of experimental trials fixating on the target (children: M = .74 SD = 0.18, adults: M = .87 SD = 0.20) and cue (children: M = .58 SD = 0.20, adults: M = .71 SD = 0.25). Additionally, adults spent a lesser proportion of experimental trials fixating on distractors (children: M = .08 SD = 0.08, adults: M = .04 SD = 0.12).

Age in months for children was not found to significantly correlate with cue fixation score $r(39)$ = .17, p = .303, target fixation score $r(39)$ = .08, p = .605, or distractor fixation score $r(39)$ = -.29, p = .066. The size of the congruency effect was found to significantly correlate

with both target fixation *r*(39) = -.53, *p* < .001 and distractor fixation *r*(39) = .83, *p* < .001, but not cue fixation *r*(39) = -.27, *p* = .099, in children.

2.2 HTKS scores

We did not find a significant correlation between HTKS score and the size of the congruency effect observed in response times for our child participants *r*(39) = .11, *p* = .491.

2.3 Error trials

Child participants made comparatively more errors (*M* = 4.96%, *SD* = 0.07) than adults (*M* = 0.05%, *SD* = 0.01). Across age groups, participants made relatively fewer fixations to the cue (error: *M* = .56 *SD* = 0.35, accurate: *M* = .66 *SD* = 0.33) and to the target (error: *M* = .66 *SD* = 0.31, accurate: *M* = .82 *SD* = 0.27), and relatively more fixations to the distractor (error: *M* = .14 *SD* = 0.20, accurate: *M* = .05 *SD* = 0.15) in error trials. To investigate the association between errors and eye movements, we conducted a paired-samples t-test on child participants. Children who made at least one error on experimental trials made significantly more fixations to the target *t*(29) = 2.62, *p* = .014 and significantly less fixations to the distractor *t*(29) = -4.21, *p* < .001 in accurate experimental trials.

Section 3: Guide to building and using the button-box

4.1 Background

The development of open-source electronics with easy-to-use hardware and software has allowed many researchers, including those with little to no prior knowledge of electronics and programming, to construct simple instruments for use in research.

For example, USB-based microcontrollers (e.g., Teensy, Arduino) can be wired with push buttons and programmed as a simple keyboard device for use in recording responses in cognitive tasks. Commonly used software in psychological research, such as E-Prime©, are

typically compatible with keyboard input. These devices can therefore be readily incorporated into existing program designs. Components can be acquired at very low cost, and easily assembled to suit a wide range of experimental designs and response configurations. Moreover, with no licensing or software constraints, these devices can be used with open-source packages such as PsychoPy.

The construction of a basic response box can be divided into three steps.

The first step is the construction of the box itself. Premade enclosures can be used. These typically come flat-packed with simple instructions on how to put them together. The top panel of these enclosures can be modified to fit any button configuration. Premade enclosures typically comprise most of the cost involved in creating a response box of the type outlined here, with prices varying depending on the size, materials, and brand. Researchers aiming to minimize expenses could construct a similar enclosure from scratch.

The second step involves wiring the electrical components. The level of electrical work required to wire the button box is comparable with that found in consumer-level electrical hobbyist kits. Many universities provide access to resources that allow for basic training on electronics and facilities to carry out this type of work. Where this is not available, online communities provide a valuable source of information and guidance, and the basic tools required for the level of electrical work can be acquired at a minimal cost.

The third step requires the programming of the microcontroller to carry out its required functions. Guidance on this can be found on the manufacturer's website as well as various online communities and platforms. Programming a microcontroller as a keyboard device is entry-level and requires very little prior knowledge or experience. Existing code can be used or modified as required.

In the following, we describe a simple method for constructing and programming a button-box capable of capturing response dynamics typically observed with more complex optical or electromagnetic motion capture equipment. The button-box comprises three simple push buttons wired to microcontrollers, which record the press and release of each button. The button-box can be connected to a computer using the built-in USB cable, and is compatible with any software capable of receiving input from a keyboard. Our design can be easily adapted to include more response options or configurations, making it an accessible and adaptable solution for researchers looking to design their own purpose-built response apparatuses.

4.2 A guide to construction

This response box was developed using a Teensy microcontroller and behaves as a 3- key keyboard. A Takashi CF-series sloped enclosure was fitted with three white Sanwa pushbuttons, and programmed using open-source software, Arduino IDE 1.8.9. A built-in library allows for keyboard functions to be readily programmed into the Teensy board, enabling the buttons to send “keystrokes” to a computer via the microcontroller board’s native USB port. An open-source third party library, Bounce2 (https://github.com/thomasfredericks/Bounce2) was also used. *Note: Bouncing occurs when any two metal contacts in an electronic device, such as a button, make contact and generate a signal. Due to their mechanical nature, a single button press or release can yield multiple signals. To control for this, debouncing software is often used to ensure one button or key press/release results in one signal being sent and registered by the computer.*

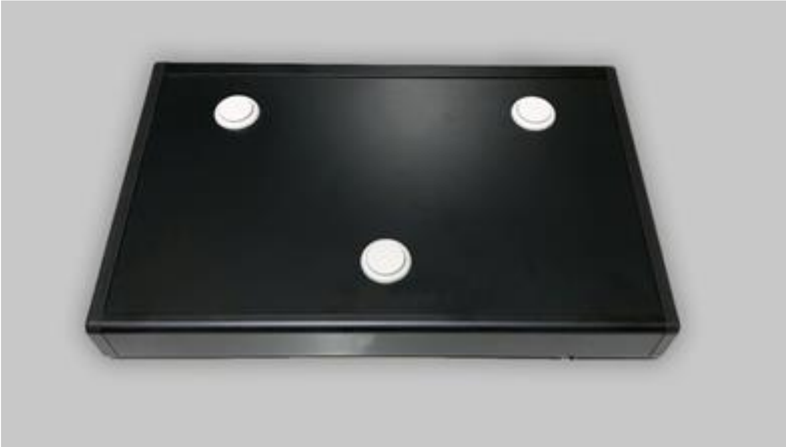

Figure 1. *Completed response box*

Components

- Teensy++ 2.0
- Takashi CF-series sloped enclosure

  Note: We originally used model CF36-23BB, however this has been discontinued at the time of writing.
- 3x Sanwa OBSF-30 Microswitch Snap-in Button (Single pole, single throw momentary switch)
- USB2.0 Type Micro B Male to Type A Male Cable

Other Materials

- Solid 20- or 22-gauge electrical wire
- Soldering iron and solder
- *Wire strippers*
- *Diagonal pliers*
- *Drill*

Step 1: Enclosure

1. Mark the locations for each button on the top panel of the enclosure
2. Drill a 30mm hole in each location and insert the buttons so that the connects of the buttons align left and right
3. Drill a hole large enough for the USB cable to pass through on either a side panel or back panel of the enclosure
4. Build enclosure as per manufacturer instructions, leaving off the top panel and one side panel

Step 2: Electrical Wiring

1. Cut six wires, each roughly 20cms long. Strip each end.
2. Solder a wire to pins 2, 4, and 6, and ground on the teensy board.
   Note: Buttons typically require a current limiting resister to function. However, Teensy microcontrollers can be programmed to have their pins connected to an internal pull-up resistor, so an external resistor is not required as part of the circuit.
3. Keeping the buttons in the top panel, solder:
   a. Wire from pin 2 to the left leg of Right button
   b. Wire from pin 4 to the left leg Centre button
   c. Wire from pin 6 to the left leg of the left button
   d. Wire from ground to the right leg of the right button
4. To complete the grounding connections, solder:
   a. A wire connecting the right leg of the right button to the right leg of the centre button
   b. A wire connecting the right leg of the centre button to the right leg of the left button.
5. Thread the USB cable through the hole and plug it into the teensy microcontroller.
6. Slide the top panel into place, and attach the side panel
   a. Note: you may want to secure the Teensy board to the top of the panel with hot glue, so that wires don't become loose when the box is being transported. It may be best to do this once the board is programmed and you are certain everything is working as it should.

Step 3: Programming

7. Download and install Arduino IDE.
8. Connect the Teensy via the USB
9. Open ResponseBox.ino code
   a. To open the code, go to File>Open… and select ResponseBox.ino. Alternatively, copy and paste the code below.
10. Select the correct settings for the Teensy++ 2.0:
   a. Tools > Board: Teensy++ 2.0
   b. Tools > USB type: Keyboard + Mouse + Joystick
   c. Tools > CPU Speed: 16Mhz
   d. Tools > Keyboard Layout: US English
11. Verify code
   a. Click the tick box on the top left, or go to Sketch > Verify/Compile

12. Upload code
    a. Select the right facing arrow on the top left, or go to Sketch > Upload
    b. A small window with a picture of the teensy board will appear, select Operation > Program. When the code is uploaded, it will show a message saying “download complete”.
13. Open a notepad and test the buttons, making sure the letters are matched to the correct keystrokes (“r” for the right button, “l” for the left button, SPACE for centre)

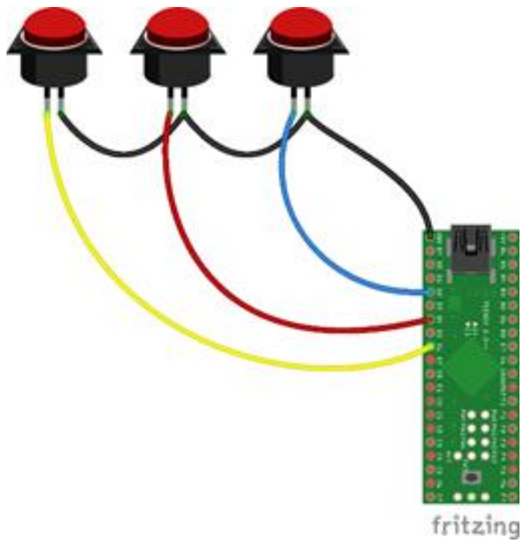

Figure 2. *Cartoon illustration of response box wiring*.

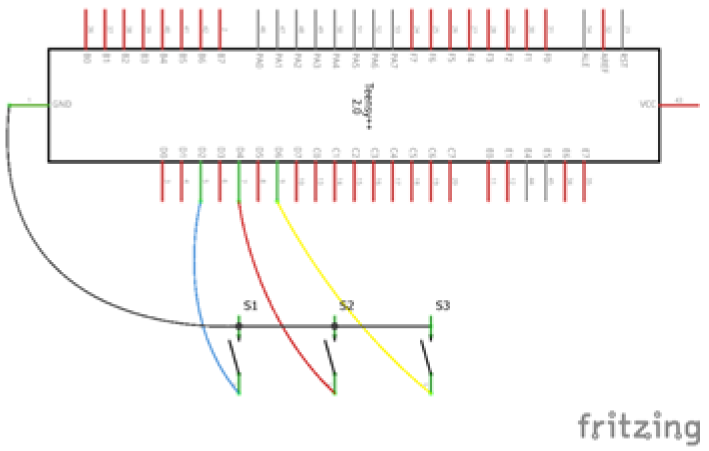

Figure 3. *Schematic of response box wiring*

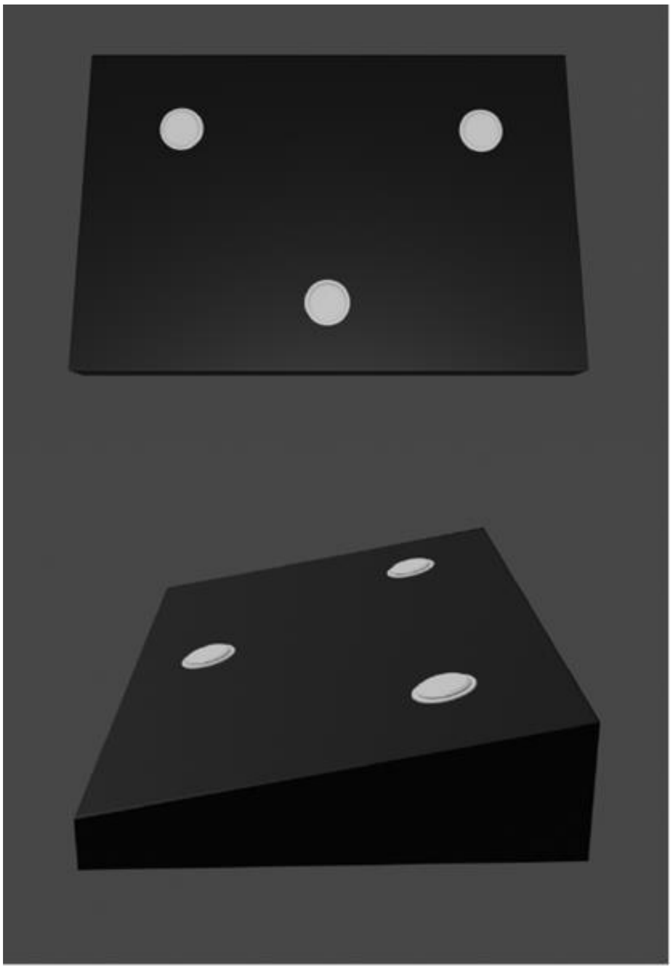

Figure 4. *3D rendering of the completed response box*

4.3 Code for programming

```
/*
 DESCRIPTION
 ====================
The following code can be used to program a simple 3-button response box using a Teensy++ 2.0
micro controller. Internal pull-up resistors are used for each button.
Bounce 2 library is used to filter mechanical noise from the buttons. The Debouncer function
introduces a timing threshold that a signal must meet for the Teensy++ 2.0 to register it as a
button/key press or release. We used a threshold of 5ms, such that the button must be pressed for
5ms for the signal to be processed as a button press/release and sent to the PC.
 */


// Include the Bounce2 library found here :
// https://github.com/thomasfredericks/Bounce2
#include <Bounce2.h>
```

```
#define BUTTON_PIN_RIGHT 2
#define BUTTON_PIN_CENTRE 4
#define BUTTON_PIN_LEFT 6

// Instantiate a Bounce objects
Bounce debouncerR = Bounce();
Bounce debouncerC = Bounce();
Bounce debouncerL = Bounce();

void setup() {

  // Setup the buttons with an internal pull-up:
  pinMode(BUTTON_PIN_RIGHT,INPUT_PULLUP);
  pinMode(BUTTON_PIN_CENTRE,INPUT_PULLUP);
  pinMode(BUTTON_PIN_LEFT,INPUT_PULLUP);

  // After setting up the buttons, setup the Bounce instance for each button:

  // Right Button:
  debouncerR.attach(BUTTON_PIN_RIGHT);
  debouncerR.interval(5); // interval in ms
  // Centre Button:
  debouncerC.attach(BUTTON_PIN_CENTRE);
  debouncerC.interval(5); // interval in ms
  // Left Button:
  debouncerL.attach(BUTTON_PIN_LEFT);
  debouncerL.interval(5); // interval in ms
}

void loop() {
```

```
// Update the Bounce instance:
  debouncerR.update();
  debouncerC.update();
  debouncerL.update();

// Get the updated value:
  int valueR = debouncerR.read();
  int valueC = debouncerC.read();
  int valueL = debouncerL.read();

// Register and send key press/release as determined by the state of the button (low = button is pressed, high = button is not pressed):
// Right Button:
        if (valueR == LOW ) {
                Keyboard.press(KEY_R);
        }
        else if (valueR == HIGH) {
                Keyboard.release(KEY_R);
        }
// Centre Button:
        if (valueC == LOW ) {
        Keyboard.press(KEY_SPACE);
        }
        else if (valueC == HIGH) {
        Keyboard.release(KEY_SPACE);
        }
// Left Button:
        if (valueL == LOW ) {
        Keyboard.press(KEY_L);
        }
        else if (valueL == HIGH) {
```

```
        Keyboard.release(KEY_L);
        }
}
```

Section 4: Analyses of log-transformed data

Table 1. Log Transformed Data

ANOVA results for all dependent variables that yielded p values < .10, for archival purposes. We adopt an alpha level of .05 throughout.

		F	η_p^2	p	Predicted Effect	Match?
**Response Time (RT)**	ci	5.01	0.05	= .028		
	CI	241.10	0.73	< .001		
	SR	38.32	0.30	< .001		
	Age	322.42	0.79	< .001		
	CI*Age	46.02	0.34	< .001		
	SR*Age	24.01	0.21	< .001		
	ci*CI	32.08	0.27	< .001		
	ci*SR	6.12	0.06	= .015		
	ci*CI*Age	12.72	0.13	= .001		
	Children					
	ci	4.48	0.09	= .040		
	CI	131.45	0.75	< .001		
	ci*CI	24.07	0.35	< .001		
	Adult					
	CI	358.37	0.89	< .001		
	ci*CI	9.56	0.18	= .003		
	ci*CI*SR	12.22	0.12	= .001		
	Alternation					
	ci	12.12	0.12	< .001	c < i	YES
	CI	133.34	0.60	< .001	C < I	YES
	Repeat					
	CI	158.19	0.64	< .001		
	ci*CI	38.02	0.30	< .001	cC < iC < iI < cI	YES
**Initiation Time (IT)**	ci	23.14	0.21	< .001	c < i	YES
	CI	161.74	0.65	< .001	C < I	YES
	SR	23.85	0.21	< .001		
	Age	295.82	0.77	< .001		
	CI*Age	33.07	0.27	< .001		
	SR*Age	12.37	0.12	= .001		
	ci*CI	10.44	0.11	= .002		
	CI*SR	4.63	0.05	= .034		
	ci*CI*Age	7.43	0.08	= .008		
	Children					
	ci	8.03	0.15	= .007		
	CI	92.65	0.68	< .001		
	ci*CI	10.14	0.19	= .003		
	Adults					
	ci	46.20	0.51	< .001		
	CI	152.33	0.78	< .001		
**Movement Time (MT)**	ci	6.86	0.07	= .010		
	CI	107.34	0.55	< .001		
	SR	4.83	0.05	= .031		
	Age	32.99	0.27	< .001		
	CI*Age	15.62	0.15	< .001		
	SR*Age	5.55	0.06	= .021		
	ci*CI	28.45	0.24	< .001		
	ci*SR	5.81	0.06	= .018		
	CI*SR	5.19	0.06	= .025		
	ci*CI*Age	7.36	0.08	= .008		
	Children					
	CI	56.54	0.56	< .001		
	ci*CI	19.13	0.30	< .001		
	Adults					
	ci	10.94	0.20	= .002		
	CI	109.01	0.71	< .001		
	ci*CI	11.16	0.20	= .002		
	ci*CI*SR	18.59	0.17	< .001		
	Alternation					
	CI	70.92	0.44	< .001	C < I	YES
	Repeat					

ci	11.10	0.11	= .001		
CI	89.55	0.50	< .001		
ci*CI	45.88	0.34	< .001	cC = iC < iI < cI	YES

Note. dfs = 88 for all analyses of adults and children combined and 44 for analyses by age group (adults and children separately).ci = Previous Congruency, CI = Current Congruency, SR = Response Type (Alternation vs. Repeat). Predicted effects were based on the results of Erb and Marcovitch (2018).

Section 5: Analyses of raw data

Table 2. Raw Data

ANOVA results for all dependent variables that yielded p values < .10, for archival purposes. We adopt an alpha level of .05 throughout.

		F	η_p^2	*p*	Predicted Effect	Match?
**Response Time**	ci	0.54	0.01	= .465		
	CI	98.32	0.53	< .001		
	SR	33.08	0.27	< .001		
	Age	193.24	0.69	< .001		
	CI*Age	45.41	0.34	< .001		
	SR*Age	27.22	0.24	< .001		
	ci*CI	21.25	0.19	< .001		
	ci*SR	6.15	0.07	= .015		
	ci*CI*Age	13.64	0.13	< .001		
	Children					
	CI	72.83	0.62	< .001		
	ci*CI	15.52	0.26	< .001		
	Adult					
	CI	362.67	0.89	< .001		
	ci*CI	6.41	0.13	= .015		
	ci*CI*SR	9.37	0.10	= .003		
	Alternation					
	ci	6.33	0.07	= .067	c < i	YES
	CI	79.35	0.47	< .001	C < I	YES
	Repeat					
	CI	103.44	0.54	< .001		
	ci*CI	30.69	0.54	< .001	cC < iC < iI < cI	YES
	ci*CI*SR*Age	4.46	0.05	= .038		
**Initiation Time**	ci	8.75	0.09	= .004	c < i	YES
	CI	84.33	0.49	< .001	C < I	YES
	SR	21.35	0.20	< .001		
	Age	230.75	0.72	< .001		
	CI*Age	43.36	0.33	< .001		
	SR*Age	15.29	0.15	< .001		
	ci*CI	12.02	0.12	< .001		
	ci*CI*Age	10.32	0.10	= .002		
	Children					
	CI	67.92	0.61	< .001		
	ci*CI	8.34	0.16	= .006		
	Adults					
	ci	43.77	0.50	< .001		
	CI	128.62	0.75	< .001		
**Movement Time**	ci	5.07	0.05	= .027		
	CI	42.67	0.33	< .001		
	SR	7.36	0.08	= .008		
	Age	33.59	0.28	< .001		
	CI*Age	17.66	0.17	< .001		
	SR*Age	7.58	0.08	= .007		
	ci*SR	7.95	0.08	= .006		
	ci*CI*Age	10.10	0.10	= .002		
	Children					
	CI	29.04	0.40	< .001		
	ci*CI	13.53	0.24	< .001		
	Adults					
	ci	13.37	0.23	< .001		
	CI	102.69	0.70	< .001		
	ci*CI	7.17	0.14	= .010		
	ci*CI*SR	15.13	0.15	< .001		
	Alternation					

CI	38.57	0.31	< .001	C < I	YES
Repeat					
ci	10.77	0.11	= .001		
CI	38.43	0.30	< .001		
ci*CI	35.51	0.29	< .001	cC = iC < iI < cI	YES

Note dfs = 88 for all analyses of adults and children combined and 44 for analyses by age group (adults and children separately). ci = Previous Congruency, CI = Current Congruency, SR = Response Type (Alternation vs. Repeat). Predicted effects were based on the results of Erb and Marcovitch (2018).
